# Gene-by-Crisis Interaction for Optimism and Meaning in Life: The Effects of the COVID-19 Pandemic

**DOI:** 10.1007/s10519-021-10081-9

**Published:** 2021-09-13

**Authors:** Lianne P. de Vries, Margot P. van de Weijer, Dirk H. M. Pelt, Lannie Ligthart, Gonneke Willemsen, Dorret I. Boomsma, Eco J. C. de Geus, Meike Bartels

**Affiliations:** 1 Department of Biological Psychology, Vrije Universiteit Amsterdam, Van der Boechorststraat 7, 1081 BT Amsterdam, The Netherlands; 2 Amsterdam Public Health Research Institute, Amsterdam University Medical Centres, Amsterdam, The Netherlands

**Keywords:** Optimism, Meaning in life, Pandemic, COVID-19, Lockdown, Heritability

## Abstract

The corona virus disease 2019 (COVID-19) pandemic and the restrictions to reduce the spread of the virus has had a large impact on daily life. We investigated the individual differences in the effect of the COVID-19 pandemic and first lockdown on optimism and meaning in life in a sample from the Netherlands Twin Register. Participants completed surveys before (N = 9964, Mean age: 48.2, SD = 14.4) and during the first months of the pandemic (i.e. April–May 2020, N = 17,464, Mean age: 44.6 SD = 14.8), with a subsample completing both surveys (N = 6461, Mean age T1: 48.8, SD = 14.5). We applied genetic covariance structure models to twin data to investigate changes in the genetic architecture of the outcome traits due to the pandemic and the interaction of genes with the environmental exposure. Although 56% and 35% of the sample was negatively affected by the pandemic in their optimism and meaning in life, many participants were stable (32% and 43%) or even showed increased optimism and meaning in life (11% and 22%). Subgroups, specifically women, higher educated people, and people with poorer health, experienced larger negative effects. During the first months of the pandemic, slightly lower heritability estimates for optimism and meaning in life (respectively 20% and 25%) were obtained compared to pre-pandemic (respectively 26% and 32%), although confidence intervals overlap. The lower than unity genetic correlations across time (.75 and .63) suggest gene-environment interactions, where the expression of genes that influence optimism and meaning in life differs before and during the pandemic. The COVID-19 pandemic is a strong exposure that leads to imbalanced effects on the well-being of individuals. Some people decrease in well-being, while others get more optimistic and consider their lives as more meaningful during the pandemic. These differences are partly explained by individual differences in genetic sensitivity to extreme environmental change. More knowledge on the person-specific response to specific environmental variables underlying these individual differences is urgently needed to prevent further inequality.

**Supplementary Information:**

The online version contains supplementary material available at 10.1007/s10519-021-10081-9.

## Introduction

The corona virus disease 2019 (COVID-19) quickly evolved into a global pandemic that had an enormous impact on people’s everyday lives. During the first year of the pandemic, there was no effective cure and vaccination for the disease and prolonged action and restrictions were needed to control the virus. In the Netherlands, the government introduced an ‘intelligent’ lockdown on 12 March 2020, to keep the virus under control, protect vulnerable groups, and make sure the healthcare system could cope with increasing demands. Major restrictions were social distancing, closing of schools, offices, and other public places, and individuals were strongly advised to work from home. As in many countries, people’s daily life and society were severely impacted. Parents had to home-school children, entire groups of people were facing financial problems or unemployment, and social contact was limited, leading to increased loneliness (Groarke et al. 2020; Killgore et al. 2020).

Although effective to reduce the spread of COVID-19, the continued restrictions in combination with distress about the virus have an impact people’s mental health and well-being. Most research on the effects of the pandemic on mental health and well-being focused on detrimental effects averaged across large population samples. Across the world, increased anxiety and depression, and decreased well-being (i.e. life satisfaction and positive affect) has been reported in response to the pandemic and first lockdown (Ahmed et al. 2020; Kwong et al. 2020; Lades et al. 2020; Ueda et al. 2020; Zacher and Rudolph 2020; Prati and Mancini 2021).

Average effects across large groups do not necessarily mean that everyone experienced negative effects but can hide potentially large individual differences. For example, Newby et al. (2020) reported increased depression and anxiety during the pandemic in around half of the Australian sample, i.e. implying that not everyone experienced an increase. Some individuals remained stable or even improved in mental health during the pandemic. Under certain circumstances people can experience psychological improvement after acute adversity (Mancini 2019). An explanation for this effect during the pandemic could be the forced pause from a busy life, less fear of missing out, and time to focus on social connections (Mancini 2020).

### Optimism and meaning in life

Aspects of well-being on which the COVID-19 pandemic can be expected to differently impact individuals are optimism and meaning in life. During the pandemic, the daily life of individuals radically changed and uncertainty about the future regarding the virus and lockdown, (e.g., When is an effective vaccine distributed worldwide? When are all restrictions lifted?), and the personal future (e.g., Will I keep my job?) increased.

Optimism is defined as the tendency to expect positive outcomes in any situation, and is related to physical and mental health and well-being (Scheier and Carver 1985, 1992, 1993; Rasmussen et al. 2009). People differ in their level of optimism and due to increased uncertainty during the pandemic individual differences may increase.

Meaning in life can be defined as people's beliefs that their lives are significant and purposeful, a feeling of achieving meaningful goals, and a sense of fulfilment (Reker and Wong 1988; Steger 2012). Feelings of meaningfulness are important for mental health, a lack is associated with psychopathology and poor well-being (Glaw et al. 2017). The lockdown can lead to a loss in meaning in life for some individuals, especially if they lose their job or have to work from home, whereas others adapt quickly to the situation and find new ways of meaningfulness.

Optimism and meaning in life are associated with each other and with other aspects of well-being, including life satisfaction (Krause 2003; Steger et al. 2006; Ho et al. 2010).

### Sources of individual differences

To understand individual differences in optimism and meaning in life, earlier research investigated the genetic and environmental influences. Twin studies have shown that around 30% of the individual differences in optimism is accounted for by genetic factors (i.e. the heritability), whereas 70% is explained by environmental influences (see van de Weijer et al. 2020). The heritability of meaning in life is estimated between 33 and 52%, with the remaining variance explained by non-shared environmental factors (Steger et al. 2011; Wang et al. 2017).

Heritability is estimated as the proportion of total variance in a phenotype that is accounted for by genetic variance.

Genetic variance may vary as a function of context and because heritability is a ratio, that depends on the total variance, it can also vary when environmental variance changes. If the environmental variance increases and the genetic variance is the same, the total variance increases and the heritability is lower. The COVID-19 pandemic can be regarded as a rare universal exposure but lockdown will impact individuals depending on how their work and home setting is affected. Some individuals could see their job degraded to ‘non-crucial’ or lose their job, whereas others see their status elevated but have to deal with high workloads. The response to lockdown thus can have differential effects on the environmental variance, as well as depend on the genetic makeup of the individual. Here, genetic variance depends on environmental exposures. For example, experimental stress can lead to new genetic variance being expressed, as well as an increase in the effects of genes influencing a trait before stress exposure (De Geus et al. 2007). The pandemic, being a rather extreme environmental stressor, can influence the genetic variance underlying optimism or meaning in life through both mechanisms. To assess the genotype by exposure interaction, a genetically informative design (e.g. twin study) that assesses participants before and during the exposure is needed. The parameters of interest are the differences in environmental and genetic variance between the two assessments (reflecting quantitative gene-environment interaction) and the genetic correlation between the measures (reflecting qualitative gene-environment interaction, with different genes being important before and during the exposure).

Several other aspects can moderate the effect of the pandemic on optimism and meaning in life. According to a systematic review (N = 19 studies), the mental health of women and younger participants was more affected during the pandemic compared to men and older participants (Xiong et al. 2020). The effect of education attainment was inconsistent; depending on the study, higher or lower educated people were more affected in their mental health during the pandemic (Daly et al. 2020; Xiong et al. 2020). Finally, people with underlying health conditions, such as chronic respiratory problems, heart problems, and autoimmune problems, have a higher risk of becoming severely ill from COVID-19 (Williamson et al. 2020) and might experience lower well-being than healthy people, out of fear or uncertainty related to attracting the virus.

We investigated the effect of the COVID-19 pandemic on optimism and meaning in life in the Netherlands during the beginning of the pandemic and the first lockdown and determinants of individual differences in these effects. We included variables that can moderate the pandemic effect: sex, age, level of education, self-rated health, and the presence of chronic illnesses. To further understand the individual differences in optimism and meaning in life before and during the pandemic, we applied longitudinal twin models.

## Method

### Sample

Participants were voluntarily registered at the Netherlands Twin Register, established by the Department of Biological Psychology, Vrije Universiteit Amsterdam (Ligthart et al. 2019). The NTR sample is a population-wide sample consisting of twins and their relatives. Every two/three years since 1991, longitudinal survey data about lifestyle, personality, psychopathology, and well-being are collected.

For a pre-pandemic measure of optimism and meaning in life, we combined data from two assessments. Just before the COVID-19 pandemic, NTR participants (i.e. twins and their families) were invited to complete a survey including the Flourishing scale (Diener et al. 2010), with an optimism and meaning in life item. This data collection stopped in February 2020 due to the pandemic. To increase the size and representativeness of the sample, we combined this sample with a sample that completed the Flourishing scale three years earlier. The samples were similar in mean and standard deviations of optimism (*M*_1_ = 5.8, *SD*_1_ = 1.1 vs *M*_2_ = 5.7, *SD*_2_ = 1.1) and meaning in life (*M*_1_ = 5.7, *SD*_1_ = 1.1 vs *M*_2_ = 5.5, *SD*_2_ = 1.1). This combined pre-pandemic sample consist of 9964 participants. To test if the timing of the pre-pandemic data has an influence on the results, we repeated the analyses separately for the two samples (i.e. just before the pandemic and three years earlier). The results can be found in the supplementary material and the associations did not change much compared to the combined pre-pandemic sample as reported in the main results.

During the pandemic and lockdown, NTR participants were invited to complete an online COVID survey and 18,035 participants returned this questionnaire. The lockdown in the Netherlands started on the 12th of March. All questionnaires were completed between 21 April 2020 and 2 May 2020. To focus on the effects of the pandemic and not of the disease, participants with a positive result on the most reliable COVID-19 test, the polymerase chain reaction (PCR) test, or an expected COVID-19 diagnosis based on the Menni model (see Blokland et al. 2020) (N = 571) were excluded, leaving a sample of 17,464 participants. Longitudinal data (i.e. pre-pandemic and pandemic questionnaire data) were present for 6461 participants.

For the genetic covariance structure modelling of longitudinal data, we analysed the data from a subset of the sample consisting of twins. For the pre-pandemic sample, we only included twin pairs that completed the pre-pandemic survey at the same time point. If the twin pairs did not have pre-pandemic data at the same time point, we only included the twin with the most recent data. The total subsample consisted of 8056 twins (either from complete or incomplete twin pairs), with 4178 MZ twins and 3878 DZ twins (see Table 1 for more details on the samples).

**Table 1:** Descriptive statistics for the samples

				Optimism		Meaning in life	
	N (females/males)	Age (SD)	Range	M (SD)	Range	M (SD)	Range
*Total sample*							
Pre-pandemic	9964 (6832/3130)	48.2 (14.4)	16–102	5.7 (1.1)	1–7	5.6 (1.1)	1–7
Pandemic	17,464 (12,391/5068)	44.6 (14.8)	16–95	7.0 (1.4)	1–10	7.5 (1.5)	1–10
Longitudinal	6461 (4532/1928)	T1: 48.8 (14.5)	16–102	5.7 (1.1)	1–7	5.6 (1.1)	1–7
		T2: 50.1 (14.1)	16–92	7.0 (1.3)	1–10	7.6 (1.4)	1–10
*Twins*							
Pre-pandemic	3879 (2719/1159)	T1: 43.2 (15.8)	16–92	5.7 (1.1)	1–7	5.5 (1.1)	1–7
Pandemic	6505 (4702/1799)	T2: 36.0 (15.1)	16–90	7.0 (1.4)	1–10	7.3 (1.6)	1–10
Longitudinal	2560 (1845/721)	T1: 44.6 (16.1)	16–102	5.7 (1.1)	1–7	5.5 (1.1)	1–7
		T2: 46.3 (15.4)	16–92	7.0 (1.4)	1–10	7.4 (1.5)	1–10

### Measures

#### Optimism

Pre-pandemic optimism was assessed with a single item of the Flourishing scale (Diener et al. 2010). Participants had to rate the item “I am optimistic about my future” on a Likert scale from 1 (strongly disagree) to 7 (strongly agree).

During the pandemic, optimism was assessed with the question “How optimistic are you about the future at the moment?”. Participants had to answer on a Likert scale ranging from 1 (not at all) to 10 (very much).

#### Meaning in life

Pre-pandemic meaning in life was assessed with an item of the Flourishing scale (Diener et al. 2010). Participants rated the item “I lead a purposeful and meaningful life” on a Likert scale from 1 (strongly disagree) to 7 (strongly agree).

In the pandemic survey, meaning in life was assessed with a single question “How meaningful do you think your life is right now?”. Participants answered on a Likert scale ranging from 1 (not at all) to 10 (very much).

#### Level of education

The level of education (i.e. highest educational attainment) of a participant was based on the longitudinal assessment of educational level. In different NTR surveys, level of education was measured with the question “What is the highest educational level that you have completed?”. The answer categories varied per questionnaire. The level of education was recoded in four categories: primary education only (1), lower vocational school and lower secondary school (2), intermediate vocational school and intermediate or higher secondary school (3) and higher vocational school and university (4).

#### Presence of chronic illnesses

To assess the presence of chronic illnesses, participants were, in both surveys, presented with a list of illnesses and were asked to indicate if they had been diagnosed with one or more illnesses. We included the following diseases: cardiovascular disorders, heart infarcts, stroke, lung diseases (e.g. asthma), liver disorders, kidney disorders, type I and type II diabetes, auto-immune disorders, cancer, neurological disorders such as dementia or Parkinson’s disease, spleen problems, and joint inflammation. When participants indicated that none of these illnesses was present they received a score of 0, if one or more of these illnesses was present, they scored a 1.

#### Self-rated health

In both surveys, self-rated health was measured with the item ‘In general, how would you rate your health?’. Participants had to answer on a 5-point Liker scale from 1 (bad) to 5 (excellent).

### Statistical analysis

#### Overall pandemic effects

To investigate the overall effect of the pandemic on optimism and meaning in life, we created a within-person change score for the subsample of participants who completed both the pre-pandemic and pandemic survey. Since the response scales were not equal, we had to rescale the pre-pandemic measure with a scale from 1–7 to a scale from 1–10 to match the pandemic measures (using the “rescale” function of the R package “scales” v0.4.1). Although the results should be interpreted with caution, as rescaling is not the ideal solution, this will lead to information about the effect of the pandemic on the optimism and meaning in life scores of the participants. We defined participants with a negative change score larger than 1 point as showing a decrease in optimism or meaning in life during the pandemic, whereas a positive change of more than 1 point indicates an increase. Participants within 1 point change were classified as stable in their optimism or meaning in life.

#### Effects of sex, age, level of education, and health

To test for the moderating effects of sex, age, level of education, self-rated health, and the presence of chronic illnesses on the effect of the pandemic on optimism and meaning in life, for each variable we ran three analyses, using Generalized estimating equation (GEE) in R to correct for familial relations in the data (Minică et al. 2015).

First, we investigated the effect of sex, age, level of education, self-rated health, and the presence of chronic illnesses (i.e. the predictors) on pre-pandemic scores. Second, we investigated the effect of this set of predictors on the scores during the pandemic. Finally, we investigated the effect of the predictors on the scores during the pandemic and included the pre-pandemic level, to correct for the baseline scores of optimism/meaning in life and investigate the effects of the predictors on the part that is left, i.e. the change in scores over time.

#### Longitudinal twin models

In the subset of twins, we conducted a longitudinal bivariate twin analysis in which we included the pre-pandemic and pandemic scores to examine the impact of the pandemic on the genetic architecture of pre- and pandemic optimism and meaning in life and to investigate the presence of genotype-environment interaction. Age and sex were included as covariates. Twin models are based on the difference in genetic similarity between monozygotic (MZ) and dizygotic (DZ) twin pairs. MZ twin pairs share (almost) all genes, whereas DZ twin pairs share on average half of their segregating genes. Based on this difference, the observed phenotypic variance and covariance between pre-pandemic and pandemic scores can be decomposed into genetic and environmental (co)variance components. Additive genetic variance (A) is variance explained by independent allele effects on the phenotype. Non-additive genetic variance (D) refers to interactions between alleles at the same locus (dominance) or at different loci (epistasis). Environmental variance includes a shared environment component (C) (shared by family members) and a non-shared component, the unique (person-specific) environment, including measurement error (E). The effects of C and D cannot be estimated simultaneously. Therefore, a choice for an ACE or ADE model was made based on the ratio of the cross-twin cross-trait correlations. If the MZ correlations are smaller than twice the DZ correlations, common environment (C) effects are expected and an ACE model is specified. If the MZ correlations are larger than twice the DZ correlations, dominant genetic (D) effects are expected and an ADE model is appropriate (Neale and Maes 2004).

First, phenotypic correlations, and cross-twin (cross-trait) correlations were estimated in a saturated model in OpenMx (Boker et al. 2011). Next, using a bivariate ACE/ADE model, we estimated genetic and environmental contributions to variance of the traits and to the covariance between the traits. Based on the bivariate analyses, we estimated the genetic and environmental correlations between the pre-pandemic and pandemic measures (de Vries et al. 2021). To answer the question how genes interact with the extreme environmental exposure, we first compared the amount of genetic and environmental variance before and during the pandemic (quantitative gene-environment interaction). In addition, we tested if the genetic correlation between the repeated measures in different environments could be constrained to 1. If not, the genetic correlation is significantly lower than 1, indicating evidence for a qualitative gene-environment interaction (Falconer 1952).

We tested the contribution of A and C/D and genetic correlation using the likelihood ratio test (LRT), comparing the full models to the nested submodels. The difference in minus two times the log-likelihood (− 2LL) between two nested models has a χ^2^ distribution with the degrees of freedom (df) equalling the difference in df between the models. If a p-value from the χ^2^ -test is significant, the fit of the constrained model is significantly worse than the fit of the more complex model. To verify the goodness of fit and the parsimony of the model, we use Akaike’s Information Criterion (AIC;(Akaike 1987). The lower the AIC value, the better the fit of the model relative to the number of parameters estimated. For the best fitting model, 95% confidence intervals were estimated.

To reduce the chance of false positive findings, we used a Bonferroni corrected *p*-value threshold (i.e. dividing 0.05 by the number of tests) of *p* = 0.002 in all analyses.

## Results

### Descriptive statistics

In Table 1, the descriptive statistics of the sample can be found. The samples before and during the pandemic differed significantly in proportion males and females, *t* = − 4.3, *p* < 0.001. During the pandemic, the proportion of females (71.0%) was higher than pre-pandemic (68.5%). Furthermore, the pre-pandemic sample was older (*M* = 48.1) than the pandemic sample (*M* = 44.6), *t* = 18.6, *p* < 0.001.

The scores on the pre-pandemic measures of optimism and meaning in life were relatively high with average scores of above 5.5 out of a maximum of 7. The pandemic means of optimism and meaning in life were respectively 7.0 and 7.5 out of 10. We cannot directly compare the mean scores of the pre-pandemic and pandemic measures, as the answer scales of the pre-pandemic and pandemic measures differed. The correlation between pre-pandemic and pandemic optimism was 0.36 (95%CI 0.33–0.38) and between pre-pandemic and pandemic meaning in life was 0.36 (95%CI 0.34–0.38). Optimism and meaning in life were also related, with a correlation of 0.59 (95%CI 0.58–0.61) pre-pandemic and 0.51 (95%CI 0.50–0.52) during the pandemic.

### Overall pandemic effects

To report on the proportions of participants that decreased, were stable or increased in optimism and meaning in life, we rescaled the pre-pandemic scores to match the pandemic answer scale. Table 2 contains the distribution of the change scores in the sample based on the rescaled variables and the characteristics of the three groups (i.e. decreasing, stable and increasing). For optimism, 56.3% of the sample showed a decrease in optimism during the pandemic, 32.5% of the sample remained stable, and 11.1% of the sample showed an increase in optimism during the pandemic. For meaning in life, only 34.8% showed a decrease, 43.1% was stable and 22.1% showed an increase in meaning in life compared to pre-pandemic levels.

**Table 2:** Characteristics of the subgroups that decreased, were stable, or increased in optimism or meaning in life during the pandemic

	Optimism			Meaning in life		
	Decrease	Stable	Increase	Decrease	Stable	Increase
N	3625	2097	715	2231	2760	1416
% of total sample	56.3	32.5	11.1	34.8	43.1	22.1
% females in subsample	72.8	66.4	67.4	75.0	67.0	68.8
Mean age (SD)	49.1 (13.8)	50.9 (14.4)	52.9 (14.9)	49.3 (14.5)	50.8 (14.0)	50.5 (13.5)
Education level (SD)	3.5 (0.7)	3.5 (0.7)	3.3 (0.8)	3.5 (0.7)	3.5 (0.7)	3.3 (0.8)
Mean self-rated health (SD)	4.1 (0.7)	4.1 (0.7)	3.8 (0.8)	4.1 (0.7)	4.2 (0.7)	4.0 (0.7)
% chronic illness	16.2	18.5	23.5	17.9	16.8	20.0

### Effects of sex, age, level of education, and health

**Fig. 1:**
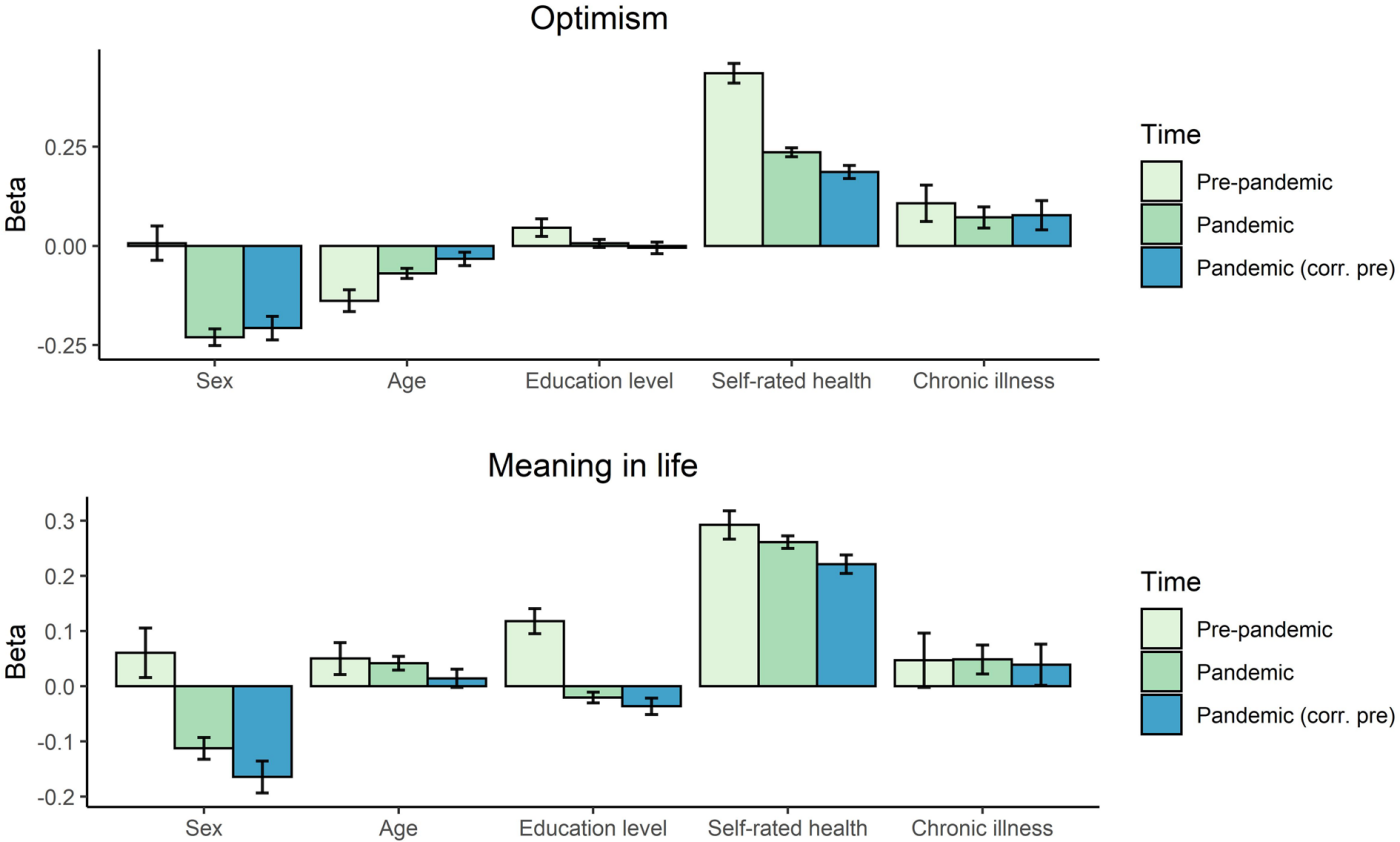
The results of the GEE analyses predicting optimism and meaning in life pre-pandemic, and during the pandemic. *Note* Pandemic (corr. pre) = the effect of the predictors on optimism/meaning in life during the pandemic when correcting for the pre-pandemic score of optimism/meaning in life

Next, we investigated the effect of sex, age, level of education, self-rated health, and the presence of chronic illnesses on optimism and meaning in life before and during the pandemic and on the change. The results can be seen in Fig. 1 and Table 3.

**Table 3:** The results of the GEE analysis on optimism and meaning in life

Optimism	Pre-pandemic		Pandemic		Pandemic	
	β (SE)	p	β (SE)	p	β (SE)	p
Sex	0.01 (.04)	0.878	− 0.24 (.02)	**3.7 × 10**^**–29**^	− 0.21 (.03)	**4.5 × 10**^**–13**^
Age	− 0.14 (.03)	**4.6 × 10**^**–07**^	− 0.07 (.01)	**8.5 × 10**^**–08**^	− 0.03 (.02)	0.074
Education	0.05 (.02)	0.038	0.01 (.01)	0.453	− 0.01 (.01)	0.693
Self-rated health	0.43 (.02)	**1.8 × 10**^**–68**^	0.23 (.01)	**8.9 × 10**^**–94**^	0.18 (.01)	**1.7 × 10**^**–27**^
Chronic illness	0.11 (.05)	0.002	0.07 (.02)	0.015	0.06 (.04)	0.082
Pre-pandemic optimism					0.30 (.01)	**6.8 × 10**^**–67**^

Meaning in life	β (SE)	p	β (SE)	p	β (SE)	p
Sex	0.06 (.05)	0.177	− 0.11 (.02)	**9.6 × 10**^**–09**^	− 0.17 (.03)	**7.9 × 10**^**–09**^
Age	0.05 (.03)	0.082	0.04 (.01)	**7.2 × 10**^**–04**^	0.01 (.02)	0.408
Education	0.12 (.02)	**1.9 × 10**^**–07**^	− 0.02 (.01)	0.026	− 0.04 (.01)	0.009
Self-rated health	0.29 (.02)	**2.2 × 10**^**–30**^	0.26 (.01)	**4.7 × 10**^**–120**^	0.22 (.01)	**2.9 × 10**^**–40**^
Chronic illness	0.05 (.05)	0.337	0.05 (.03)	0.071	0.04 (.04)	0.367
Pre-pandemic meaning in life					0.33 (.01)	**4.4 × 10**^**–78**^

Bolded *p*-values are significant at *p* < 0.002

#### Optimism

Pre-pandemic, there were no effects of sex and level of education on optimism (see Table 3). Men (*M* = 5.74, *SD* = 1.09) reported similar optimism levels compared to women (*M* = 5.73, *SD* = 1.05). There was a small effect of age on optimism. The older the participants, the lower the level of optimism. Better self-rated health was associated with a higher optimism level. Finally, the slightly higher optimism of participants without a chronic illness (*M* = 5.77, *SD* = 1.04) compared to participants with an illness (*M* = 5.44, *SD* = 1.21) did not reach significance (*p* = 0.002).

During the pandemic, an effect of sex on optimism appeared. Women (*M* = 6.88, *SD* = 1.35) reported lower levels of optimism than men (*M* = 7.20, *SD* = 1.36). There was still an effect of age on optimism with older participants reporting lower optimism and an effect of self-rated health, with higher self-rated health being associated with higher optimism. Again, the higher optimism of participants without chronic illness (*M* = 7.00, *SD* = 1.35) compared to with a chronic illness (*M* = 6.82, *SD* = 1.42) did not reach significance.

When including pre-pandemic optimism as predictor, there was a significant effect of pre-pandemic optimism, sex, and self-rated health on pandemic optimism. The positive effect of pre-pandemic optimism indicates that higher pre-pandemic optimism is associated with a higher level of optimism during the pandemic as well. The effect of sex indicates that, when correcting for baseline optimism, being female compared to male was associated with lower optimism during the pandemic. The same applies to self-rated health, correcting for the baseline effect, a poorer self-rated health was associated with lower optimism compared to a better self-rated health during the pandemic.

#### Meaning in life

Pre-pandemic, there was no effect of sex and age on meaning in life (see Table 3). Men (*M* = 5.58, *SD* = 1.08) and women (*M* = 5.59, *SD* = 1.08) reported a similar level of meaning in life. There was a significant relation between level of education and meaning in life. Participants with a higher level of education reported a higher meaning in life. A better self-rated health was related to a higher meaning in life, whereas there was no effect of the presence of chronic illnesses.

During the pandemic, an effect of sex on meaning in life appeared. Women (*M* = 7.42, *SD* = 1.52) reported a lower meaning in life during the pandemic than men (*M* = 7.64, *SD* = 1.48). There was a small but significant effect of age on meaning in life, with older participants reporting higher meaning in life. The effect of education level on meaning in life was not significant anymore. Better self-rated health was still associated with a higher meaning in life. There was no effect of the presence of chronic illnesses.

When including pre-pandemic meaning in life as predictor, there was a significant effect of pre-pandemic meaning in life, sex, and self-rated health on pandemic meaning in life. The positive effect of pre-pandemic meaning in life indicates that a higher pre-pandemic meaning in life is associated with a higher level of meaning in life during the pandemic as well. The other effects indicate that when correcting for baseline meaning in life, being female compared to male was associated with a lower in meaning in life during the pandemic and poorer self-rated health was associated with lower in meaning in life compared to a better self-rated health.

### Longitudinal twin models

The twin correlations of optimism and meaning in life are reported in Table 4. Both pre-pandemic and during the pandemic, the monozygotic twin correlations were higher than the dizygotic twin correlations, suggesting genetic influences on optimism and meaning in life at both times. The cross-twin cross-trait correlations were also higher for MZ than for DZ twins, indicating an influence of genetic effects on the association between the pre-pandemic and pandemic measure for optimism and meaning in life. As there was no evidence for dominant genetic effects (i.e. r_mz_ < 2 * r_dz_), we specified ACE models.

**Table 4:** Cross-trait cross-twin correlations for pre-pandemic and pandemic optimism and meaning in life

Optimism	MZ		DZ	
	Pre-pandemic	Pandemic	Pre-pandemic	Pandemic
Pre-pandemic	0.25 (.18–.32)		0.15 (.01–.28)	
Pandemic	0.16 (.11–.22)	0.20 (.13–.26)	0.13 (.03–.22)	0.09 (.01–.17)

Meaning in life	Pre-pandemic	Pandemic	Pre-pandemic	Pandemic
Pre-pandemic	0.31 (.23–.38)		0.23 (.10-.34)	
Pandemic	0.17 (.12–.23)	0.26 (.20–.31)	0.12 (.04–.21)	0.09 (.01–.18)

#### Optimism

**Fig. 2:**
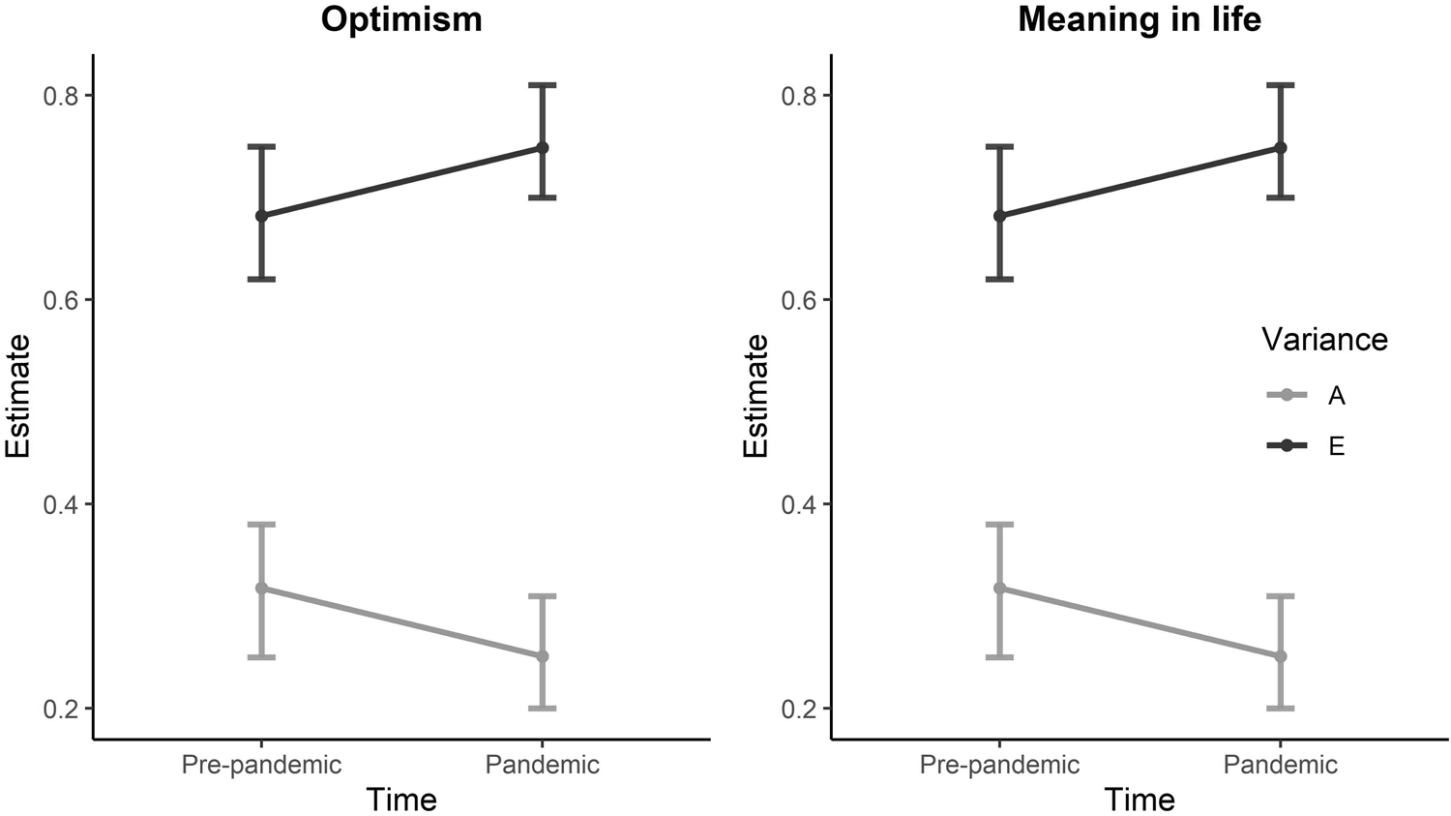
The standardized estimates for genetic and environmental variance underlying optimism and meaning in life. *A* additive genetic variance, *E* environmental variance

The model fitting showed that dropping the shared environmental component did not lead to a significant change in model fit (*p* = 0.841) (see Supplementary Table S3). Dropping the additive genetic component instead of the shared environmental component did also not lead to a change in fit (*p* = 0.119). However, as the lower AIC suggests that the AE model is the best fitting model, the standardized estimates of the AE model are reported in Table 5 and Fig. 2. In supplementary Table S1, the standardized estimates of the full ACE model are reported.

**Table 5:** Standardized estimates for additive genetic and nonshared environmental influences on pre-pandemic and pandemic optimism and meaning in life and their covariance based on the best fitting models

Optimism	A		E	
	Pre-pandemic	Pandemic	Pre-pandemic	Pandemic
Pre-pandemic	0.26 (.19–.32)		0.74 (.68–.81)	
Pandemic	0.49 (.34–.64)	0.20 (.14–.25)	0.51 (.36–.66)	0.80 (.75–.86)

Meaning in life	A		E	
	Pre-pandemic	Pandemic	Pre-pandemic	Pandemic
Pre-pandemic	0.32 (.25–.38)		0.68 (.62–.75)	
Pandemic	0.44 (.31–.56)	0.25 (.20–.31)	0.57 (.45–.69)	0.75 (.70–.81)

*A* standardized additive genetic effects, *E* standardized non-shared environmental effects

The standardized estimates indicate that before the pandemic 26% (95%CI 19–32%) of the individual differences in optimism was explained by additive genetic effects, whereas the remaining 74% (95%CI 68–81%) was explained by unique environmental influences. During the pandemic, the heritability estimate was 20% (95%CI 14–25%), with the remaining 80% (95%CI 75–86%) of the variance explained by unique environmental effects.

The bivariate heritability was 49% (95%CI 34–64%), whereas 51% (95%CI 36–66%) of the covariance of optimism across the two measurements was explained by environmental effects. The genetic and environmental correlations between optimism pre-pandemic and during the pandemic were respectively 0.75 (95%CI 0.53–0.98) and 0.23 (95%CI 0.15–0.30). Based on the 95% confidence interval, the genetic correlation was different from 1. Also constraining the genetic correlation between the pre-pandemic and pandemic measure of optimism to 1 resulted in a poorer model fit, although this did not reach our significance threshold, *p* = 0.037. The potential lower than unity correlation suggests qualitative gene-environment interaction effects with partly different genes for optimism pre-pandemic and during the pandemic.

#### Meaning in life

For meaning in life, the model fitting (see Supplementary Table S3) showed that dropping the shared environmental component did not lead to a change in fit (*p* = 0.523). Dropping the additive genetic component also did not lead to a change in fit (*p* = 0.010). However, the lower AIC suggests the AE model is the best fitting model. In Table 5 and Fig. 2, the standardized estimates of the AE model can be found (see Supplementary Table S2 for the standardized estimates of the full ACE model).

Before the pandemic, 32% (95%CI 25–38%) of the individual differences in meaning in life was explained by additive genetic effects, whereas the remaining 68% (95%CI 62–75%) was explained by unique environmental influences. During the pandemic, 25% (95%CI 20–31%) of the individual differences in meaning in life was explained by additive genetic effects, with the remaining 75% (95%CI 70–81%) explained by unique environmental effects.

The bivariate heritability was 44% (95%CI 31–56%), whereas 57% (95%CI 45–69%) of the covariance of meaning of life across the two measurements is explained by environmental effects. The genetic and environmental correlation between meaning in life pre-pandemic and during the pandemic was respectively 0.63 (95%CI 0.47–0.80) and 0.33 (95%CI 0.25–0.39). Based on the 95% confidence interval, the genetic correlation was different from 1. Constraining the genetic correlation between the pre-pandemic and pandemic measure of meaning in life to 1 resulted also in a poorer model fit, *p* = 4.0 × 10^–5^. The lower than unity correlation suggests a qualitative gene-environment interaction effect with partly different genes for meaning in life pre-pandemic and during the pandemic.

## Discussion

We report individual differences in the effect of the pandemic and first lockdown in the Netherlands on two aspects of well-being: optimism and meaning in life. In line with previous research a substantial part of the sample showed a decrease in optimism and meaning in life during the pandemic (respectively 48.9% and 28.4%). However, around half of the sample showed stability in optimism and meaning in life and the remaining 11% and 22% of the sample even increased compared to pre-pandemic levels. These results indicate that not everyone experienced reduced well-being in response to the pandemic and lockdown. An explanation for this stability or increase in well-being in these unprecedented times could be the forced pause from a stressful, busy life and more time to focus on social connections during the pandemic (Mancini 2020).

To further investigate the individual differences in well-being in response to the pandemic, we tested moderation of the response by sex, age, level of education and health. A consistent finding was the lower optimism and meaning in life levels of women compared to men during the pandemic, whereas before the pandemic women and men reported similar levels. This sex effect is consistent with other research that reported the mental health of women to be more affected during the pandemic compared to men (Pieh et al. 2020; Solomou and Constantinidou 2020; Xiong et al. 2020). An explanation could be the different burden for women versus men during the lockdown (Alon et al. 2020). As schools and daycare closed during the lockdown, the caregiving responsibilities of families increased. Mothers have reduced their working hours more than fathers (Alon et al. 2020; Collins et al. 2020), and reported more transitions to working from home and reductions in working hours (Reichelt et al. 2020), indicating an unequal distribution of caregiving responsibilities within families. To explore the effect of caregiving responsibilities of men and women on optimism and meaning in life during the pandemic in our sample, we conducted an exploratory analysis on the interaction effect of being male or female and having children in the household (none, children > 12 and children < 12). In a subsample of around 9000 participants, using GEE, there was no main effect of children in the household (p = 0.88 and p = 0.09) or interaction effect with sex (p = 0.15 and p = 0.23) on optimism and meaning in life respectively. This indicates that the higher optimism and meaning in life for men compared to women is unlikely to be explained by caregiving responsibilities in our sample.

Additionally, the sex difference could result from the negative effect of the pandemic on the health care, retail and service industry, in which women are overrepresented. Especially health care workers report high levels of anxiety, depression, and insomnia during the COVID-19 pandemic (Pappa et al. 2020). Particularly in nurses (predominantly female), depression and anxiety scores were increased compared to doctors, as nurses may experience a greater risk of COVID-19 exposure by providing more direct care to patients.

The level of education influenced the effect of the pandemic on meaning in life. While pre-pandemic there was a clear association between meaning in life and educational attainment, this generally higher meaning in life for higher educated participants disappeared during the pandemic. Participants that decreased in meaning in life during the pandemic were higher educated, whereas participants that increased were lower educated. Explanations for this result in earlier studies included the higher news consumption and greater concerns about COVID-19 of higher educated people (Daly et al. 2020). Furthermore, during the pandemic a larger number of higher educated people are faced with stressors and demands that lower educated people are more likely to have experienced before, e.g., experiences of job instability and childcare difficulties. Finally, higher educated people might experience larger changes in daily life, as higher educated people are more likely to be forced to work from home due to pandemic measurements, whereas lower educated people are more likely to perform practical work in the workplace that often continued under the difficult pandemic circumstances.

Lastly, self-rated health had the expected effect on optimism and meaning in life during the pandemic. As people with a poorer health are at risk for severe COVID-19 (Xiong et al. 2020) they are also more likely to be negatively affected in their well-being.

### Genetic and environmental influences

The twin modelling indicated a slightly lower heritability and larger relative effect of the environmental factors during the pandemic than pre-pandemic, but confidence intervals overlap. This relatively stable heritability estimate indicates no evidence for quantitative gene-environment interaction effects and thus a stable influence of genetic effects before and during the pandemic. Alternatively, the pandemic and lockdown could have led to more variance in person-specific environmental variables such as the working situation (i.e. some people have lost their job, whereas others have to work more), home situation (i.e. living alone, with a partner or with children) and caregiving responsibilities (i.e. home schooling children), resulting in increase in total variance and the slight decrease in heritability.

The high genetic (0.75 and 0.63 for optimism and meaning in life) correlations between the pre-pandemic and pandemic measures indicate a large overlap in the genetic factors underlying the traits at both time points. These high genetic influences are in line with the high genetic correlations between pre-pandemic and pandemic measures in a range of psychological and behavioral traits as reported by Rimfeld et al. (2021). However, although strong, the correlations are significantly different from unity, providing an indication for possible qualitative gene-environment interaction effects. Different genes express their influence under different environmental circumstances, in this case in response to the pandemic.

The environmental correlations were substantially lower (0.23 and 0.33 for optimism and meaning in life), indicating that the environmental factors at both time points are mostly unique to that time point, as could be expected due to the strong environmental impact of the pandemic. We note that E also includes measurement error and that the two constructs were assessed by a single item. Finally, the bivariate heritability between the pre-pandemic and pandemic measure is respectively 49% and 44% for optimism and meaning in life, indicating that around half of the association between the measures can be explained by genetic factors.

### Implications, limitations and future directions

Although the lockdown in the first wave of the COVID-19 pandemic was effective to supress the spread of the virus in the Netherlands, the measures did affect people’s optimism and meaning in life. In line with previous research, we report that a substantial part of the sample showed a decrease in the well-being variables optimism and meaning in life during the pandemic. However, the largest part of the sample showed stable levels or even increased levels of optimism and meaning in life during the pandemic. Special attention is needed for specific subgroups of the population (i.e. women, and people with a poorer health), as they are especially at risk for negative effects. Finally, possible qualitative gene-environment interaction underline the pandemic as a strong environmental variable.

A limitation of the current study was the different response scales for the pre-pandemic and pandemic measures and the slightly different wording of the items, limiting the possibilities to directly compare the scores. To be able to create within-subject change scores and report an average effect of the pandemic, we rescaled the pre-pandemic scores (1–7) to the answer scale of the pandemic measures (1–10). This solution has limitations and is not ideal. Therefore, we only used the within-subject scores to report on the proportion of people that are stable, increased or decreased and did not further investigate the subgroups showing a decrease or increase during the pandemic.

Furthermore, for both optimism and meaning in life we used single item measures at both time points. Whereas single item measures are time efficient, there is discussion about the decreased reliability compared to multiple item questionnaires (e.g. Bowling 2005; Diamantopoulos et al. 2012), although in the field of well-being, the reliability of single item measures have been reported to be adequate and only slightly lower compared to the reliability of multiple item measures (Krueger and Schkade 2008; Jovanović and Lazić 2020).

In addition, the findings of the study might be influenced by the representativeness of our sample for the Dutch population. The subset of NTR participants in this particular study included more females (around 70%) than males and was relatively highly educated, with more than 50% indicating having attended higher vocational school or university, while in the Dutch population this percentage is around 35% (CBS, Enquête Beroepsbevolking (EBB), 2020). Also when comparing the pre-pandemic and pandemic sample, there might be a response bias. For example, the pandemic sample (*M* = 44.6) was younger than the pre-pandemic sample (*M* = 48.1).

The pandemic data analysed in this study was from the beginning of the pandemic (i.e. April and May 2020). During these months the pandemic and lockdown were novel and people did not know how long the pandemic and lockdown would last. Many countries, including the Netherlands experienced multiple waves of COVID-19 infections and a prolonged lockdown. Comparing the effect of the first and the second lockdown on mental health, increased depressive symptoms and a higher psychological burden in the second lockdown were reported in Germany and Austria, although the restrictions were less strict compared to the first lockdown (Moradian et al. 2021; Dale et al. 2021). Therefore the results for optimism and meaning in life might also be different further into the pandemic or the effects might change over time. People’s well-being might be more affected or genetic sensitivity might play a larger role later during the pandemic or in a later lockdown. For example, the optimism of people might decrease when the pandemic lasts longer or certain people might find another way to experience meaning in life. Therefore, in future research, the longitudinal effects of the pandemic and prolonged lockdown on the genetics of well-being should be investigated.

Another direction for future research is the specific environmental variables that lead to differences in well-being between subgroups and individuals, such as the working situation or care-giving responsibilities. We indicated a large group of people that were stable or showed an increase in optimism and meaning in life during the pandemic. This group is worth studying in more detail to get a hold of possible protective factors and resilience mechanisms. More knowledge on the person-specific response to individual specific environmental variables underlying the differences between people is urgently needed to prevent further inequality. Furthermore, to prevent the decrease of well-being in the subgroups, targeted policies and help are needed.

To conclude, the pandemic is a strong environmental variable that leads, due to genetic differences between people, to imbalanced effects on well-being of individuals. More knowledge on the person-specific response to specific environmental variables underlying these individual differences is needed to prevent further inequality. Therefore, we urge for more research to inform the development of programs to prepare individual well-being and health systems for future pandemics in different areas of the world.

## Supplementary Information

Below is the link to the electronic supplementary material.

Supplementary file1 (DOCX 21 kb)

Supplementary file2 (XLSX 59 kb)

## Data Availability

The data from the Netherlands Twin Register may be accessed through the Netherlands Twin Register (ntr.fgb@vu.nl) upon approval of the data access committee.
