# Supplementary material for: Gene-by-Crisis Interaction for Optimism and Meaning in Life: The Effects of the COVID-19 Pandemic
Source: Behav Genet. 2021 Sep 13;52(1):13–25. doi: 10.1007/s10519-021-10081-9 (PMC8437088; doi:10.1007/s10519-021-10081-9)
Supplement: Supplementary file 1 — Supplementary file1 (DOCX 21 kb) [file 10519_2021_10081_MOESM1_ESM.docx]

**Supplementary Tables**

Table S1. Standardized estimates for the full ACE twin model for optimism

|  | **A** |  | **C** |  | **E** |  |
| --- | --- | --- | --- | --- | --- | --- |
|  | Pre-pandemic | Pandemic | Pre-pandemic | Pandemic | Pre-pandemic | Pandemic |
| Pre-pandemic | 0.20 |  | 0.05 |  | 0.75 |  |
|  | (-.09-.52) |  | (-.24-.31) |  | (.68-.82) |  |
| Pandemic | 0.23 | 0.21 | 0.24 | -0.014 | 0.53 | 0.80 |
|  | (-.39-.85) | (-.01-.42) | (-.32-.80) | (-.19-.16) | (.37-.69) | (.74-.86) |
|  |  |  |  |  |  |  |
| Correlation | 0.37 |  | NA |  | 0.24 |  |
|  | (NA-NA) |  |  |  | (.16-.31) |  |

**Note:** A= standardized additive genetic effects, C= standardized shared environmental effects, E = standardized environmental effects.

Table S2. Standardized estimates for the full ACE twin model for meaning in life

|  | **A** |  | **C** |  | **E** |  |
| --- | --- | --- | --- | --- | --- | --- |
|  | Pre-pandemic | Pandemic | Pre-pandemic | Pandemic | Pre-pandemic | Pandemic |
| Pre-pandemic | 0.17 |  | 0.14 |  | 0.69 |  |
|  | (-.11-.46) |  | (-.13-.39) |  | (.62-.77) |  |
| Pandemic | 0.25 | 0.33 | 0.17 | -0.07 | 0.58 | 0.74 |
|  | (-.23-.74) | (.13-.54) | (-.27-.60) | (-.25-.10) | (.45-.71) | (.69-.80) |
|  |  |  |  |  |  |  |
| Correlation | 0.44 |  | NA |  | 0.33 |  |
|  | (NA-NA) |  |  |  | (.26-.40) |  |

**Note:** A= standardized additive genetic effects, C= standardized shared environmental effects, E = standardized environmental effects.

Table S3. Model fitting results for the longitudinal twin models for optimism and meaning in life before and during the pandemic

|  | Model | Base | Test | ep | -2LL | df | AIC | ΔLL | Δdf | p |
| --- | --- | --- | --- | --- | --- | --- | --- | --- | --- | --- |
| **Optimism** | SAT |  |  | 17 | 17273.9 | 5360 | 6553.9 |  |  |  |
|  | 1 | SAT | ACE | 13 | 17282.8 | 5364 | 6554.8 | 5.5 | 2 | 0.063 |
|  | 2 | 1 | **AE** | **10** | **17283.6** | **5367** | **6549.6** | **0.8** | **3** | **0.841** |
|  | 3 | 1 | *CE* | *10* | *17288.6* | *5367* | *6554.6* | *5.9* | *3* | *0.119* |
|  | 4 | 1 | E | 7 | 17376.3 | 5370 | 6636.3 | 93.5 | 6 | 5.6x10^-18^ |
|  | 5 | 2 | rG=1 | 10 | 17287.9 | 5368 | 6551.9 | 4.33 | 1 | 0.037 |
|  | Model | Base | Test | ep | -2LL | df | AIC | ΔLL | Δdf | p |
| **Meaning in life** | SAT |  |  | 17 | 17906.6 | 5299 | 7308.6 |  |  |  |
|  | 1 | SAT | ACE | 13 | 17912.5 | 5303 | 7306.5 | 2.2 | 2 | 0.329 |
|  | 2 | 1 | **AE** | 10 | **17914.7** | **5306** | **7302.7** | **2.2** | **3** | **0.523** |
|  | 3 | 1 | *CE* | *10* | *17923.9* | *5306* | *7311.8* | *11.4* | *3* | *0.010* |
|  | 4 | 1 | E | 7 | 18045.3 | 5309 | 7427.3 | 132.8 | 6 | 3.3x10^-26^ |
|  | 5 | 2 | rG=1 | 10 | 17931.6 | 5307 | 7317.6 | 16.9 | 1 | 4.0x10^-5^ |

**Note**: SAT= saturated model, ep=estimated parameters, -2LL= -2*loglikelihood, df= degrees of freedom, ΔLL= change in loglikehood, Δdf= change in degrees of freedom.
